# Curcumin Prevents Cerebellar Hypoplasia and Restores the Behavior in Hyperbilirubinemic Gunn Rat by a Pleiotropic Effect on the Molecular Effectors of Brain Damage

**DOI:** 10.3390/ijms22010299

**Published:** 2020-12-30

**Authors:** Silvia Gazzin, Matteo Dal Ben, Michele Montrone, Sri Jayanti, Andrea Lorenzon, Alessandra Bramante, Cristina Bottin, Rita Moretti, Claudio Tiribelli

**Affiliations:** 1 Fondazione Italiana Fegato-Onlus, Bldg. Q, AREA Science Park, ss14, Km 163.5, Basovizza, 34149 Trieste, Italy; dalben.matteo@yahoo.it (M.D.B.); mi.montrone@gmail.com (M.M.); sri.jayanti@fegato.it (S.J.); ctliver@fegato.it (C.T.); 2 SPF Animal Facility, CBM Scarl, Bldg. Q2, AREA Science Park, ss14, Km 163.5, Basovizza, 34149 Trieste, Italy; andrelorenzon@gmail.com (A.L.); alessandrabio.ab@gmail.com (A.B.); 3 Department of Medical Sciences, Ospedale di Cattinara, University of Trieste, Strada di Fiume 447, 34149 Trieste, Italy; cbottin@units.it; 4 Neurology Clinic, Department of Medical, Surgical, and Health Sciences, University of Trieste, Strada di Fiume 447, 34149 Trieste, Italy; moretti@units.it

**Keywords:** kernicterus, bilirubin, phototherapy, nutraceutic, GRAS, newborns, pre-term, inflammation, redox, glutamate neurotoxicity

## Abstract

Bilirubin toxicity to the central nervous system (CNS) is responsible for severe and permanent neurologic damage, resulting in hearing loss, cognitive, and movement impairment. Timely and effective management of severe neonatal hyperbilirubinemia by phototherapy or exchange transfusion is crucial for avoiding permanent neurological consequences, but these therapies are not always possible, particularly in low-income countries. To explore alternative options, we investigated a pharmaceutical approach focused on protecting the CNS from pigment toxicity, independently from serum bilirubin level. To this goal, we tested the ability of curcumin, a nutraceutical already used with relevant results in animal models as well as in clinics in other diseases, in the Gunn rat, the spontaneous model of neonatal hyperbilirubinemia. Curcumin treatment fully abolished the landmark cerebellar hypoplasia of Gunn rat, restoring the histological features, and reverting the behavioral abnormalities present in the hyperbilirubinemic rat. The protection was mediated by a multi-target action on the main bilirubin-induced pathological mechanism ongoing CNS damage (inflammation, redox imbalance, and glutamate neurotoxicity). If confirmed by independent studies, the result suggests the potential of curcumin as an alternative/complementary approach to bilirubin-induced brain damage in the clinical scenario.

## 1. Introduction

If exposed to a high level of bilirubin, a metabolite of hemoglobin, the CNS may undergo serious and permanent damage, especially when the challenge occurs in neonatal life. Classical symptoms are hearing disability, cognitive impairment, loss of voluntary movements, and communication, recently recapped by the term kernicterus spectrum disorder (KSD) [1]. Neonatal hyperbilirubinemia is a common condition, affecting more than 60% of full-term and 80% of preterm neonates [2,3]. Its proper management requires immediate recognition and an effective therapeutical approach, with phototherapy (PT, reducing bilirubin challenging by its oxidation) representing the most used one. Nevertheless, KSD continues to be a challenge everywhere [4,5,6]. In developed countries, cases of neurological consequences of bilirubin neurotoxicity are reported both in neonates exposed to PT [7,8], as well in subjects with total serum bilirubin below the known risk threshold [9,10,11]. Still, in developed regions, the emerging population of pre-term infants is at higher risk of developing KSD, not only due to its intrinsic fragility but also because PT seems to be less efficient in decreasing serum free bilirubin (Bf, the real fraction of blood bilirubin entering the brain) [12,13,14]. Despite the delay or even the absence of screening playing a major role in KSD in low–medium income countries [15,16,17], unavailability or inefficiency of PT is a co-morbidity that still condemns many newborns to permanent neurological damage or even death [6]. Indeed, recent warnings about the safety of aggressive use of PT have been raised [18,19,20,21], and exchange transfusion and albumin infusion, the current existing alternative therapeutical approaches, are even riskier. Thus, we believe there is a need for improving the therapeutical options [4,22].

Decades of investigation on the molecular events undergoing bilirubin toxicity to the brain described the involvement of thousands of genes, proteins, and signaling pathways (as an example but the list is much longer [22,23,24,25,26,27,28,29,30,31,32,33,34,35,36,37,38,39,40,41]). Inflammation, redox state imbalance, and glutamate toxicity have been repeatedly described and look like the ideal candidates to be targeted by drugs [22]. Based on a previous ex vivo study [22], we aimed to find a possible effective pharmacological treatment able to protect the brain without necessarily decreasing the bilirubin in the blood.

This approach has been explored in the Gunn rat, the spontaneous model for KSD [42,43,44] by the use of minocycline in the past. Unfortunately, although fully effective in rats [23,45,46] this molecule cannot be used in neonates due to its severe side-effects [5,47]. To find an alternative molecule applicable to neonates, we tested curcumin (Curc), a nutraceutical already used in the treatment of other diseases and potentially able to counteract the aforementioned damaging mechanism of bilirubin in the CNS [48,49,50,51,52,53,54,55,56,57,58]. Curc consumption is part of the culture in Nepal and in India, where it has been used not only as a spice but also as a therapeutical principle in Ayurveda, the traditional Indian medicine (reviewed in [59]). Curc is a “generally recognized as safe” (GRAS) compound with no demonstrated side effects even at high dosages by the EMA and the FDA [60]. Of relevance to our purpose is the fact that Curc can rapidly cross the blood-brain barrier, and accumulating into the brain [55,61,62,63].

Based on this background, in the present work, we explored the efficacy of Curc in counteracting the main pathological mechanism ongoing bilirubin-induced brain damage [22] in vivo, by using the Gunn rat, the well-characterized spontaneous model of neonatal hyperbilirubinemia [42,43,44,45].

## 2. Results

### 2.1. Evaluation of the Efficacy of Curcumin in Preventing the Cerebellar Damage in the Gunn Rat

**Figure 1:**
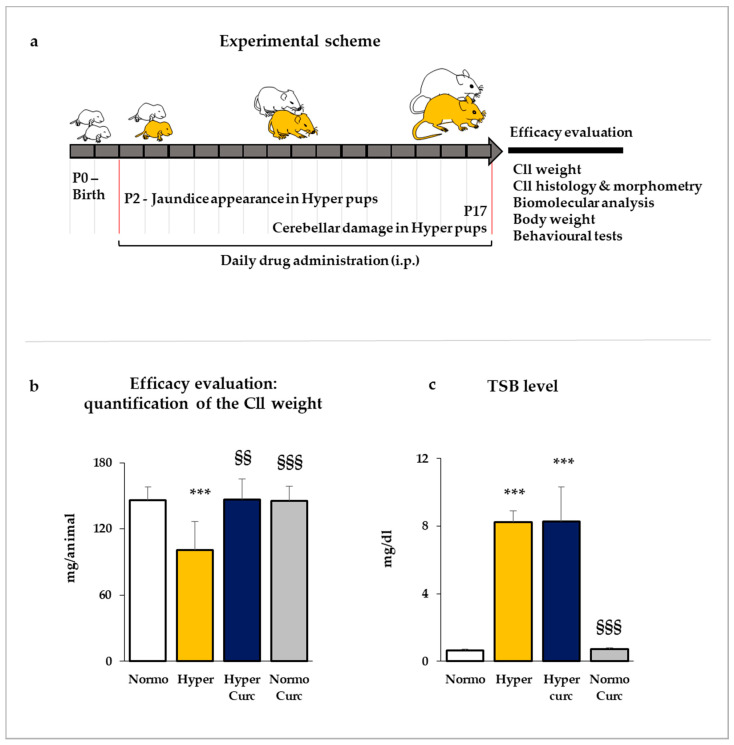
Experimental plan and efficacy of curcumin in rescuing cerebellar hypoplasia. (**a**) Two days after birth (P2: post-natal age in days), the litters composed both by normobilirubinemic (Normo—white) and hyperbilirubinemic (Hyper—yellow) pups were daily injected intraperitoneally (i.p.) with curcumin (Curc). At P17, the animals were sacrificed, and the cerebellum (Cll) was submitted for different analyses to deeply evaluate the drug efficacy and the mechanisms of action. (**b**) The Cll weight (expressed in mg/animal) in P17 pups was quantified and used as a first, immediate evaluation of the efficacy of the treatment. (**c**) Total serum bilirubin (TSB in mg/dL) was quantified in each group to monitor any potential alteration due to the treatment. Both (**b**,**c**) With white bars—untreated normobilirubinemic (Normo) Gunn pups; yellow bars—untreated hyperbilirubinemic rats (Hyper); blue bars—curcumin-treated hyperbilirubinemic pups (Hyper Curc); gray bars—curcumin-treated normobilirubinemic pups (Normo Curc). Data were expressed as mean ± S.D. In each experimental group, ≥10 animals were considered. Statistical significance was evaluated by the one-way analysis of variance (ANOVA), followed by a Tukey–Kramer Multiple Comparisons Test when *p*-value < 0.05. Statistical significance: ***: *p <* 0.001 vs. Normo pups. §§, §§§: *p <* 0.01, *p <* 0.001 vs. Hyper rats.

As depicted in Figure 1a, the treatment was started two days after the birth (P2), when jaundice appears in hyperbilirubinemic pups and was continued up to P17, when the cerebellar (Cll) hypoplasia, the reduced Purkinje cell number, and the reduced thickness of the external granular cell layer, reaches statistical significance [32,43,44,45,46,64,65,66,67,68]. The restoration of these landmark features of bilirubin-induced brain toxicity represents a major checkpoint and a major proof of the treatment efficacy.

#### 2.1.1. Evaluation of the Cll Weight

As shown in Figure 1b, in P17 hyperbilirubinemic pups (Hyper—yellow bar) the Cll weight loss reached 33% vs. the matched normobilirubinemic controls (Normo—white bar, *p* < 0.001). When Curc was administered (Hyper Curc—blue bar), complete protection against the Cll hypoplasia was observed (not statistically different from Normo; *p* < 0.01 vs. Hyper).

#### 2.1.2. Assessment of the Histological Findings of the Cll under the Curc Treatment

**Figure 2:**
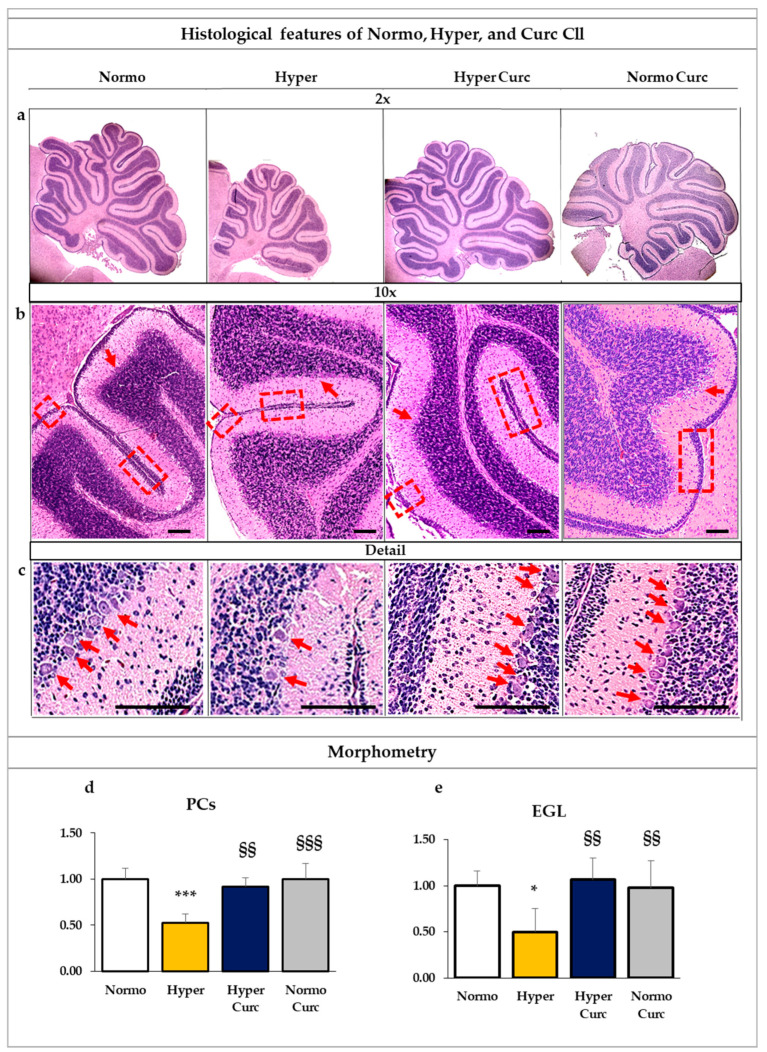
Curc prevents the alterations of the histological and morphometric features characteristic of the Hyper Gunn rat. (**a**–**c**) Histological features of the cerebellum (Cll) in Normo (normobilirubinemic), Hyper (hyperbilirubinemic), and curcumin (Hyper Curc and Normo Curc) treated P17 Gunn rats. Different magnifications are shown to allow appreciating the whole Cll details. (**a**) = 2×: Curc fully prevents the reduction in the volume of the Cll present in the Hyper P17 pups. (**b**) = 10×: allows to better appreciate the general appearance of the external granule cell layer (EGL, e.g., squares) and the Purkinje cells (PCs, e.g., arrows), both reduced in Hyper, and reverted to the physiological features by Curc. The protective effect of Curc on PC number is even more visible in (**c**) the detailed picture (e.g., arrows). Scale bar: 200 μm. (**d**,**e**) Morphometric evaluation of the PC number (**d**) and external EGL thickness (**e**). White bars: Normo; yellow bars: Hyper; blue bars: Hyper Curc; grey bar Normo Curc. Data are in mean ± S.D. and expressed as fold vs. the Normo pups used as reference (=1). Three animals of each genotype and treatment were devoted to this goal. Statistical significance was evaluated by the one-way analysis of variance (ANOVA), followed by a Tukey–Kramer Multiple Comparisons Test when *p*-value < 0.05. Statistical significance: */***: *p* < 0.05, *p* < 0.001 vs. Normo age-matched pups. §§/§§§: *p* < 0.01, *p* < 0.01 vs. Hyper age-matched pups.

To further analyze the protective effect of Curc on the Cll damage induced by bilirubin toxicity, we performed histological and morphometric analysis. The general appearance of the Cll, the number of the Purkinje neurons (PCs), as well as the thickness of the external granule cell layer (EGL), were quantified (Figure 2).

As shown in Figure 2a, 2× magnification, the reduction of the Cll volume typical of Hyper pups was fully restored in hyperbilirubinemic pups treated with Curc, in line with the restoration of the Cll weight. At higher magnification (Figure 2b = 10× and c = Detail), the reversal of the reduction of the PC number is also evident (e.g., arrows) and the reversal of the EGL thickness (e.g., rectangles) in Hyper Curc pups.

As detailed by the quantification performed on the whole Cll section (Figure 2d), the PC number was reduced by about 50% in Hyper pups vs. Normo littermates (*p* < 0.001), and fully restored by Curc (not statistically significant vs. Normo; *p* < 0.01 vs. Hyper). Similarly, as detailed by the EGL thickness graph (Figure 2e) the significant reduction in Hyper animals (50%, *p* < 0.05 vs. Normo), was reversed to the physiological level represented by the Normo pups by Curc (not statistically significant vs. Normo; *p* < 0.01 vs. Hyper).

#### 2.1.3. Total Serum Bilirubin Quantification

To assess if the efficacy of Curc in normalizing the Cll features was related to a potential decrease in the level of serum bilirubin (the stressor), we quantified the total serum bilirubin (TSB) concentration. As shown in Figure 1c, Hyper pups presented 17 times higher TSB than Normo age-matched pups (*p <* 0.001). Curc treatment did not modify the TSB (not statistically different from Hyper; *p <* 0.001 vs. Normo), indicating that the protective effect was not mediated by a reduction of the bilirubin challenging, but rather due to the in situ (Cll) interference with the main pathological mechanisms of bilirubin toxicity.

### 2.2. Behavioral Tests

**Figure 3:**
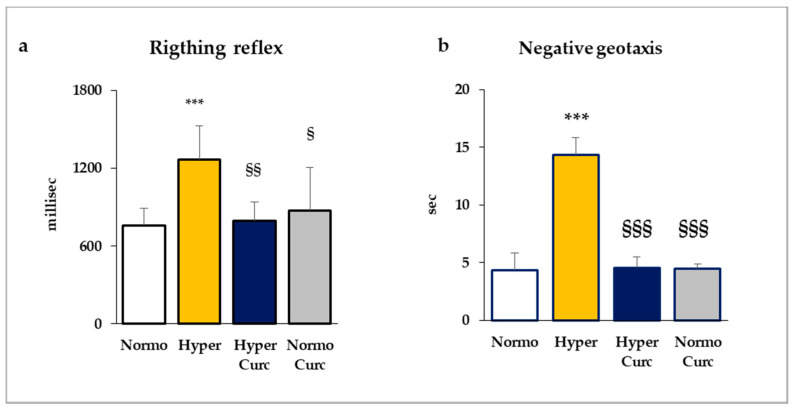
Curc restores the motor abilities of Hyper Gunn rats. The righting reflex (**a**) and the negative geotaxis (**b**) were selected as optimal tests for assessing the motor and coordination abilities of Gun rat pups. With white bars—untreated normobilirubinemic (Normo) Gunn pups; yellow bars—untreated hyperbilirubinemic rats (Hyper); blue bars—curcumin-treated hyperbilirubinemic pups (Hyper Curc); gray bars—curcumin-treated normobilirubinemic pups (Normo Curc). Data are in mean ± S.D., and in milliseconds (millisec) for the righting reflex and in seconds (sec) in the negative geotaxis test. In each experimental group, ≥3 animals were studied. Statistical significance was evaluated by the one-way analysis of variance (ANOVA), followed by a Tukey–Kramer Multiple Comparisons Test when *p*-value < 0.05. Statistical significance: ***: *p* < 0.001 vs. Normo pups. §; §§, §§§: *p* < 0.05, *p* < 0.01, *p* < 0.001 vs. Hyper rats.

As conclusive evidence of the protection from brain damage conferred by the molecule, we evaluated the behavior of the pups (Figure 3) by two tests: the righting reflex, and negative geotaxis (see detail on the material and method section).

As shown in Figure 3, Hyper pups needed a significantly longer time for finishing both the tests. In the righting reflex test (Figure 3a), while Normo pups rapidly regained the correct position, Hyper pups required about 1.7-fold more time (*p* < 0.001 vs. Normo). Hyper Curc performed as the controls (not statistically different from Normo; *p* < 0.01 vs. Hyper).

Similarly, Hyper rats were statistically less skilled (*p* < 0.001) compared to Normo littermates in the negative geotaxis test (Figure 3b). Also, in this test, the performance of Hyper pups was fully restored by Curc treatment (Hyper Curc, not statistically different from Normo; *p* < 0.001 vs. Hyper).

### 2.3. Monitoring of the Side Effects of the Treatment

**Figure 4:**
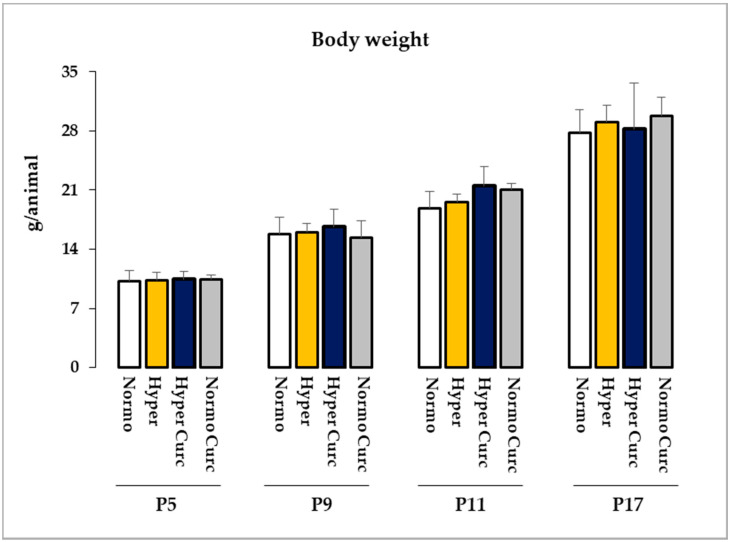
Bodyweight recording. Bodyweight in developing Gunn rats is expressed as mean ± S.D., and as g/animal. P: post-natal age in days. With white bars—untreated normobilirubinemic (Normo) Gunn pups; yellow bars—untreated hyperbilirubinemic rats (Hyper); blue bars—curcumin treated hyperbilirubinemic pups (Hyper Curc), grey bars—Curc treated Normo rats (Normo Curc). In each experimental group, ≥10 animals were studied. No statistical differences were noticed.

In the end, 14 Hyper pups and 12 Normo littermates as controls were treated with Curc. No distress at the daily monitoring [69], no decreased Cll weight (Figure 1b), no abnormal morphometric features (Figure 2), no changed TSB (Figure 1c), no abnormal behavior (Figure 3), no decreased body weight (Figure 4), no deaths were observed, indicating the absence of side effects for this formulation of Curc in this model.

### 2.4. Effects of Curc on the Main Molecular Effectors Involved in Bilirubin Brain Damage

To substantiate the ability of Curc in preventing bilirubin-induced Cll damage, we explored the modulation of selected markers of inflammation, redox imbalance, as well as glutamate release [22,23,24,25,26,27,28,29,30,31,32,33,34,35,36,37,38,39,40,41] both at gene and protein level.

#### 2.4.1. Evaluation of the Effect of Curc on the Inflammatory Markers

**Figure 5:**
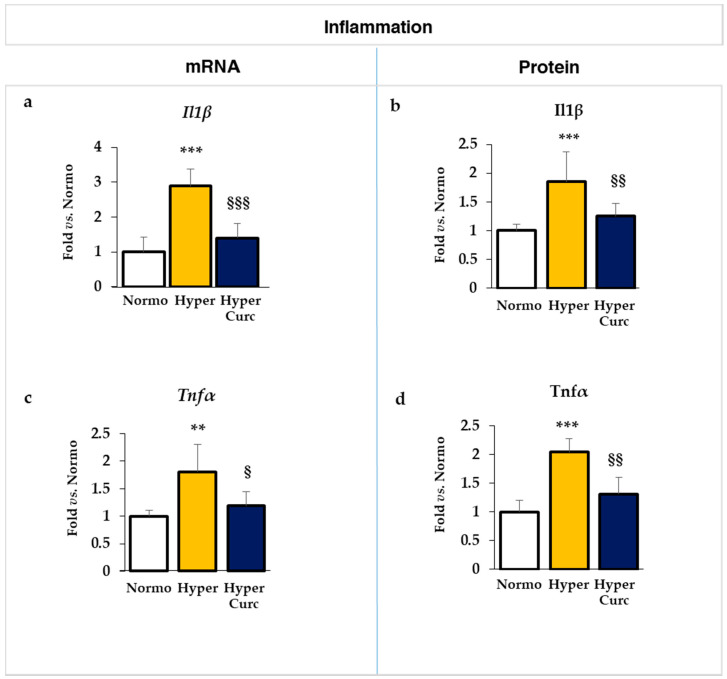
Curc prevents the induction of inflammation in the Hyper Gunn rat. The effect of Curc on the mRNA (**a,c**) and protein (**b,d**) level of *Il1β*/Il1β (interleukin 1beta—**a**,**b**) and *Tnfα*/Tnfα (tumor necrosis factor-alpha, **c**,**d**) were evaluated by RTqPCR and ELISA, respectively. With white bars—untreated normobilirubinemic (Normo) Gunn pups; yellow bars—untreated hyperbilirubinemic rats (Hyper); blue bars—curcumin-treated hyperbilirubinemic pups (Hyper Curc). Data are in mean ± S.D. and expressed as fold vs. the Normo pups used as reference (=1). In each experimental group, ≥6 animals were studied. Statistical significance was evaluated by the one-way analysis of variance (ANOVA), followed by a Tukey–Kramer Multiple Comparisons Test when *p*-value < 0.05. Statistical significance: **, ***: *p* < 0.01 and *p* < 0.001 vs. Normo pups. §; §§, §§§: *p* < 0.05, *p* < 0.01, *p* < 0.001 vs. Hyper rats.

As reported in Figure 5, the mRNA (italicized) expression of both *Il1β* (interleukin 1beta Figure 5a) and *Tnfα* (tumor necrosis factor-alpha, Figure 5c) was significantly increased in Hyper vs. Normo pups (*p* < 0.001 and *p* < 0.01, respectively). In hyperbilirubinemic rats treated with Curc, the expression of both cytokines decreased to levels comparable to the Normo controls (both not significantly different vs. Normo. *Il1β p* < 0.001 and *Tnfα p* < 0.05 vs. Hyper).

The same pattern was found at the level of the protein (Figure 5b–d, not italicized), where the two times up-regulation present in Hyper pups for both Il1β and Tnfα (both *p* < 0.001), was reversed by Curc to a level comparable with the Normo rats (1.25-fold and 1.31-fold, respectively, both not statistically significant vs. Normo. Both *p* < 0.01 vs. Hyper).

#### 2.4.2. Evaluation of the Effect of Curc on the Glutamate Cll Content

**Figure 6:**
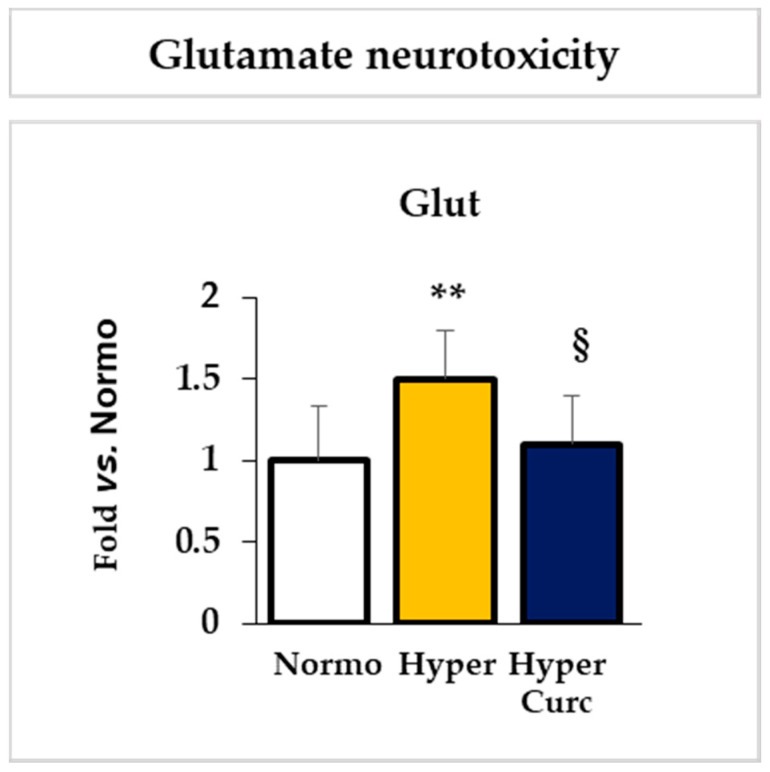
Curc prevents the increase of glutamate in the Hyper Gunn rat. The effect of Curc on the glutamate content in the Cll of hyperbilirubinemic pups was quantified by an enzymatic test. With white bars—untreated normobilirubinemic (Normo) Gunn pups; yellow bars—untreated hyperbilirubinemic rats (Hyper); blue bars—curcumin-treated hyperbilirubinemic pups (Hyper Curc). Data are in mean ± S.D. and expressed as fold vs. the Normo pups used as reference (=1). In each experimental group, ≥6 animals were studied. Statistical significance was evaluated by the one-way analysis of variance (ANOVA), followed by a Tukey–Kramer Multiple Comparisons Test when *p*-value < 0.05. Statistical significance: **: *p* < 0.01 vs. Normo pups. § *p* < 0.05 vs. Hyper rats.

As shown in Figure 6, the 1.5 times increase of the glutamate (Glut) content in the Cll of Hyper rats (*p* < 0.01), responsible for glutamate neurotoxicity, was also restored by Curc to levels found in the Normo animals (not statistically different from Normo; *p* < 0.05 vs. Hyper).

#### 2.4.3. Evaluation of the Effect of Curc on the Redox Markers

**Figure 7:**
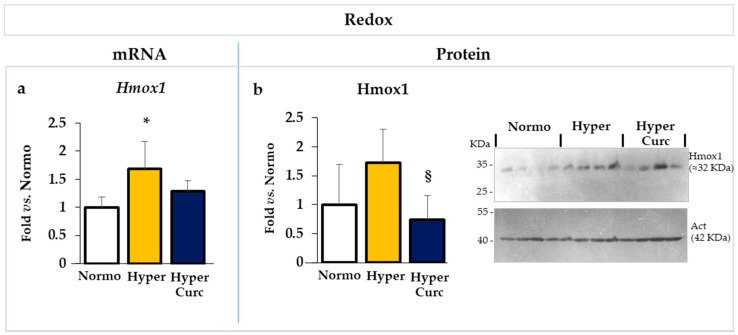
Curc prevents the increase in the redox stress in the Hyper Gunn rat. The effect of Curc on the mRNA (**a**) and protein (**b**) level of *Hmox1*/Hmox1 (heme oxygenase 1), a redox sensor, was evaluated by RTqPCR and Western blot, respectively. With white bars—untreated normobilirubinemic (Normo) Gunn pups; yellow bars—untreated hyperbilirubinemic rats (Hyper); blue bars—curcumin-treated hyperbilirubinemic pups (Hyper Curc). Data are in mean ± S.D. and expressed as fold vs. the Normo pups used as reference (=1). In each experimental group, ≥6 animals were studied. Statistical significance was evaluated by the one-way analysis of variance (ANOVA), followed by a Tukey–Kramer Multiple Comparisons Test when *p*-value < 0.05. Statistical significance: *: *p* < 0.05 vs. Normo pups. § *p* < 0.05 vs. Hyper rats.

Similar to cytokines, the mRNA expression of *Hmox1* (Heme oxygenase 1 Figure 7a), a redox sensor, was significantly up-regulated in Hyper pups (*p* < 0.05), and reverted in Hyper rats treated with Curc. At the level of the protein (Figure 7b), despite no statistical significant, a trend toward induction in Hyper pups, again, reverted by Curc was observed.

Collectively, these data support the conclusion that Curc interferes with the main pathological mechanisms undergoing bilirubin brain toxicity [22] preventing brain damage and restoring brain functions in Hyper Gunn pups.

### 2.5. Additional Evaluation of Curc Protection: Selected Markers of Brain Development

To further explore the efficacy of Curc protection, we monitored selected genes/proteins involved in brain development and maturation, whose alterations in Hyper Gunn rats have been recently suggested to participate in the progression of cerebellar hypoplasia, and that we know to be altered at P17 in Hyper pups [67].

**Figure 8:**
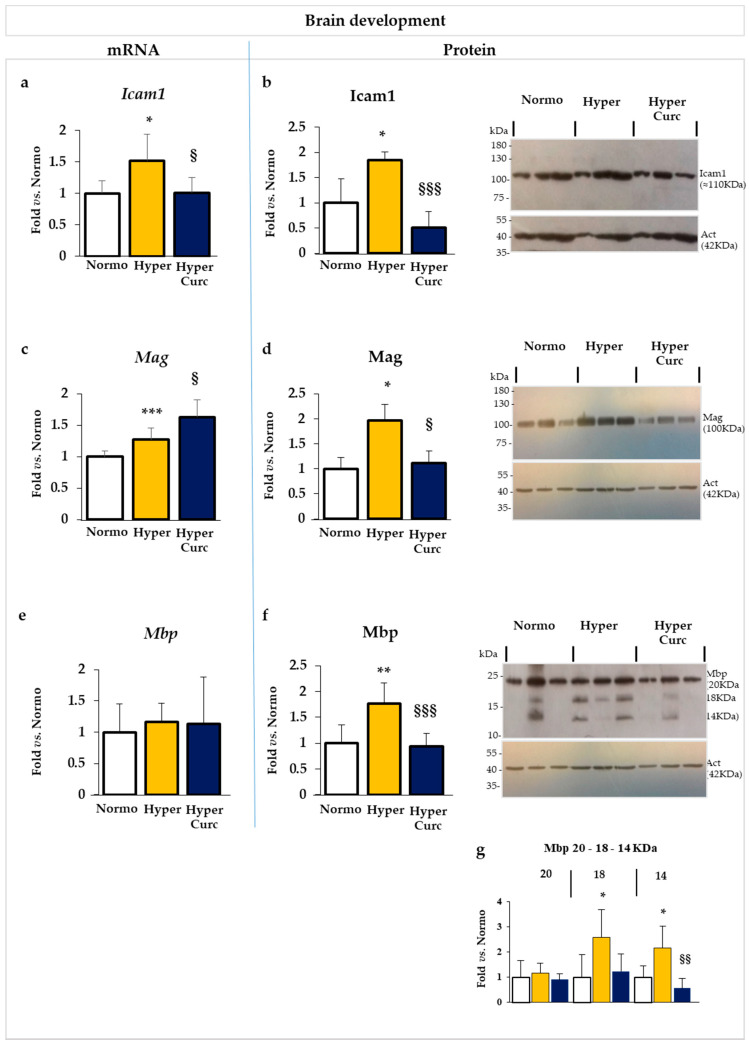
Curc normalizes the level of selected markers of brain development in the Hyper Gunn rat. The effect of Curc on the mRNA (**a**,**c**,**e**) and protein (**b**,**d**,**f**,**g**) level of *Icam1*/Icam1 (intercellular adhesion molecule 1, Figure 8a,b), *Mag*/Mag (myelin-associated glycoprotein, Figure 8c,d), and *Mbp*/Mbp (myelin basic protein, Figure 8e,f,g), known to be altered in P17 Hyper pups, were evaluated by RTqPCR and Western blot, respectively. With white bars—untreated normobilirubinemic (Normo) Gunn pups; yellow bars—untreated hyperbilirubinemic rats (Hyper); blue bars—curcumin-treated hyperbilirubinemic pups (Hyper Curc). Data are in mean ± S.D. and expressed as fold vs. the Normo pups used as reference (=1). In each experimental group, ≥6 animals were studied. Statistical significance was evaluated by the one-way analysis of variance (ANOVA), followed by a Tukey–Kramer Multiple Comparisons Test when *p*-value < 0.05. Statistical significance: *, **, ***: *p* < 0.05, *p* < 0.01, *p* < 0.001: vs. Normo pups. §, §§, §§§: *p* < 0.05, *p* < 0.01, *p* < 0.001 vs. Hyper rats.

As shown in Figure 8a,b, *Icam1*/Icam1 (intercellular adhesion molecule 1—involved in brain maturation and morphogenesis [67]) mRNA (Figure 8a) and protein (Figure 8b) levels were both significantly increased in Hyper pups (both *p* < 0.05 vs. Normo) reverting to a level comparable to controls in Curc rats.

*Mag* (myelin-associated glycoprotein: myelination, dendritogenesis, cell projection organization [67]) mRNA (Figure 8c); was only marginally increased in Hyper, but was significantly upregulated in Hyper pups treated with Curc (*p* < 0.001 vs. Normo; *p* < 0.05 vs. Hyper). Conversely, the protein level of Mag (Figure 8d) was significantly induced in Hyper pups (*p* < 0.05), fully reversing to the physiological level in rats treated with the molecule.

No relevant mRNA modulation of *Mbp* (Figure 8e) myelin basic protein: stabilization of myelin [70]) was detected neither in Hyper nor Hyper Curc rats with respect to Normo littermates. At the protein level (Figure 8f), similarly to Mag, Mbp was induced in Hyper (*p* < 0.01 vs. Normo) and restored to the physiological level by Curc (no statistical difference vs. Normo; *p* < 0.001 vs. Hyper). The up-regulation (and normalization under Curc treatment) in the protein level of Mbp in Hyper pups was due to an increase of the 18 and 14KDa bands (both *p* < 0.05 vs. Normo), while the 20KDa protein was not significantly different from Normo or Hyper Curc samples (Figure 8g: detail on Mbp quantification).

The high molecular weight (MW) isoform of Mbp is known to control the proliferation of the oligodendrocytes, while the two lower bands represent the mature myelin strongly expressed by mature oligodendrocytes in rodents [70,71]. Interesting is the good agreement with the increased level of Mag in Hyper pups, with Mag involved in myelin stabilization and repair after demyelinating injuries [72]. In this paper, we are not focusing on the mechanisms of bilirubin brain damage, nevertheless the data support our previous hypothesis [67] that Hyper pups at P17 try to react to the insult of bilirubin occurred in the post-natal development by increasing myelination. By preventing the bilirubin brain damage, Curc may not be associated with the hyper-activation of Mag and Mbp. The alteration (perturbation and recovery) of myelin production in the developing hyperbilirubinemic Gunn rat, is an interesting point that should be better addressed in the future by a devoted study.

## 3. Discussion

The need to increase the therapeutic approaches to KSD, as well as find supportive solutions when adequate medical care is not available, prompted us to evaluate a novel pharmacological approach aimed to counteract directly the main molecular mechanisms of bilirubin-induced brain damage, without necessarily decreasing TSB.

A similar approach was used in the past in the Gunn rat by the administration of minocycline [23,45,46]. Despite the drug being fully effective in protecting the rat brain from neurological damage, the side effects of minocycline prevented its clinical use [5,47]. Analogs with less/no side effects have been developed and tested, but unfortunately, the loss of toxicity of the new molecules was accompanied by the loss of the protective effect [23]. Indeed single anti-oxidant, anti-inflammatory, glutamate channel antagonist principles alone were not, or only partly, able to restore damage [23,36,73], suggesting that none of these mechanisms alone was determinant. In line with this conclusion, in preliminary work we reported that only treatment with a cocktail of anti-inflammatory and anti-oxidant drugs together with a glutamate channel blocker almost fully reverted the damage in an ex vivo setting, suggesting that bilirubin toxicity was due to a synergistic effect of different mechanisms [22].

Based on that, and to accelerate the possible clinical application to newborns, we used Curc because the molecule is already used in other diseases, because it can enter and accumulate into the brain [55,61,62,63], and because it is reported to be able to counteract all the main pathological mechanisms undergoing KSD [48,49,50,51,52,53,54,55,56,57,58,74].

The data we present support rather strongly the efficacy of the Curc formulation we used in preventing cerebellar damage (weight—Figure 1b; volume, EGL, PCs: Figure 2a–e) typical of the Hyper Gunn rats [32,44,45,46,64,65,66,67]. The protection was not mediated by lowering serum bilirubin level (Figure 1c), but rather to a direct effect on the damaging mechanisms (inflammation, glutamate excitotoxicity, and redox state—Figure 5, Figure 6 and Figure 7), with the normalization of the behavior in Hyper Curc pups (Figure 3) representing a convincing proof of the efficacy of Curc.

The results may be explained by the multiple functions of Curc, a pleiotropic capability that the molecule shares with minocycline [75]. In addition to anti-oxidant effect (e.g., on HMOX1 [50]), curcumin possesses anti-inflammatory (on IL6, COX2, TNFα, IL1β), anti-apoptotic, anti-tumorigenic effect, and acts on numerous signaling pathways [50,51,52,53,54,55,56,57]. The modulatory and protective effects of curcumin against glutamate neurotoxicity have also been previously reported [48,49,58].

Although this study did not aim to the study of the signaling pathways controlled by the drug, our data indicated that curcumin might restore *Il1β*/Il1β, *Tnfα*/Tnfα, *Hmox1*/Hmox1, and *Icam1*/Icam1 levels by acting, in an opposite way, on the same transcriptional targets of bilirubin. Differently, concerning *Mag*/Mag and *Mbp*/Mbp, the post-transcriptional mechanisms prevail in the result under Curc treatment. In sum, it looks like the final effect and signaling pathway involved might be strongly target-dependent. Further devoted studies are needed to unravel the multiple signaling pathways responsible for curcumin protection in KSD.

It should be mentioned that doubts on Curc efficacy and safety in the clinical scenario exist with the available literature equally divided into positive and negative results (see the reviews [50,52,57,76]). Curc is mainly used in cancer therapy. It has the ability to suppress cancer growth by interfering with cell division [52,54], but the reported side effects (diarrhea, headache, rash, yellow stool, nausea, increased serum alkaline phosphatase and lactate dehydrogenase, abdominal pain; usually reported at very high dosage and long exposition) call for precaution [50,57,76]. On the other side, Curc consumption up to 2–2.5 g/day (about 40 mg/kg) is part of the culture in Nepal and India, where it is used in the traditional Indian Ayurveda medicine (reviewed in [59]). Curc is a “generally recognized as safe” (GRAS) compound (EMA and FDA) with no demonstrated side effects even at high dosages [60]. The typical dosage in adults is about 4–8 g/day (about 60–114 mg/kg), and dosages up to 12 g/day (about 170 mg/kg) for several months have been reported [50,57], with minor side effects. Beneficial effects of Curc administration in infants for bowel disease are reported [59,77,78,79], and in pediatric cancer, 400 mg/day of Curc orally administered for nine months to a six-month-old baby with infantile hemangioendothelioma, has been reported as improving body weight gain, reducing liver dimensions with no evidence of residual hepatic lesions, and without reporting side effects [77]. This suggests that Curc administration in neonatal hyperbilirubinemia (for 24 h in case of PT; or 10–20 days in case of spontaneous normalization of blood bilirubin levels in absence of PT [80,81]) might be, in principle, feasible. 

Recent studies on the accumulation of Curc in the rodent brain indicate that the molecule enters rapidly and accumulates into CNS, especially when administered for a long period (chronic assumption) [61]. Importantly, no significant differences from oral vs. i.p. administration have been reported. The extrapolation of what was observed in the experimental model to the newborn still needs to be demonstrated [61].

The daily administration of the drug as soon as jaundice appeared, possibly interfering promptly with the damage, might be an additive reason for the success of the work. In this respect, we believe our experimental scheme may well reproduce the clinical scenario of a severely hyperbilirubinemic infant not treated with PT.

The dose we used (10 mg/kg) is below the posology reported in clinical trials (see above) and lower also in respect of other works in rodent models of CNS diseases [82,83,84]. This suggests that small amounts of the molecule might be effective in counteracting KSD.

Our data (Figure 1b,c Cll weight and TSB; Figure 2 histology and morphometry; Figure 3 behavior, and Figure 4 bodyweight) also agree with previous works, where benefits and no side effects of the molecule have been documented [82,83,84]. Nevertheless, independent confirmations of our results with different doses and different timing of treatment in animal models are required before suggesting Curc in the clinical setting. Efficacy/safety evaluation in the human is, in any case, an obligated step, considering a possible species-specific different sensitivity to the principle.

In conclusion, these data indicate the ability of the formulation of Curc we used in preventing the bilirubin brain induced damage in the Gunn rat model of neonatal hyperbilirubinemia. We need to confirm these data before curcumin might be considered to be administered to the jaundiced newborns. If successful, the possibility to deliver the principle diluted in the baby-bottle may become a feasible alternative/complementary approach to PT where this is not easily available.

## 4. Materials and Methods

### 4.1. Litter Composition and Treatment Scheme

Gunn rats (Hds Blue: Gunn-UDPGTj) were obtained from the SPF animal facility of CBM S.c.a.r.l. (AREA Science Park, Basovizza). Litters were obtained by mating heterozygous normobilirubinemic (Normo) females with heterozygous hyperbilirubinemic (Hyper) males. Animals were housed in a temperature-controlled environment (22 ± 2 °C), on a 12 h light/dark schedule, and ad-libitum access to food and water. The entire litter composed of both Normo and Hyper pups was used (percentage of Normo/Hyper = 51%/49%, respectively; mean number of pups in each litter: 6.6). Based on our experimental experience demonstrating that the sex is not relevant for the model, both male and female were used (final % of M/F 44%/56%, respectively). Exclusion criteria were litters with less than two Normo, less than two Hyper, or the presence of small, sick animals at the starting of the challenge.

Curcumin (Curc: Curcusoma, 10 mg/Kg, BiosLine, Padova, Italy) treatment (i.p.: intraperitoneal injection) started at P2 (post-natal age in days) when jaundice was visible in Hyper pups and was repeated each day up to P17 (Figure 1a) when the cerebellar hypoplasia in Hyper rats reached the 30% (vs. Normo) [67] and may be used as an immediate indication of the drug efficacy [46].

Animal distress was monitored daily with an objective distress score table [69]. Bodyweight, as a part of the distress evaluation, was recorded by a bench balance all long the treatment (P5, P9, P11, P17; ≥10 animals in each experimental group were evaluated). Any non-zero observation on any score parameter implied the immediate interruption of the treatment for the affected animal.

At the end (P17), animals were sacrificed by decapitation under deep anesthesia (Tiletamina + zolazepam, 35 mg/kg, i.p.), and blood and tissues were collected for analysis as previously described [32,67]. Experiments were performed according to the Italian Law (decree 87-848) and European Community directive (86-606-ECC). The maximal effort was done in respecting the 3R rule. The study was approved by the competent OPBA and by the Italian Ministry (n° 1165/2015-PR and n° 1024/2020-PR).

### 4.2. Cll Weight

To assess the efficacy of the treatment, we considered the reduction of the characteristic cerebellar hypoplasia present in the Gunn rat [32,46,67], by recording the cerebellar weight by a precision balance. Data were expressed as mean ± S.D. and as mg/animal.

### 4.3. Cll histology and Morphometry

The architecture of the cerebellum was assessed by hematoxylin-eosin staining, performed in freshly dissected, paraffin-embedded brains, as previously reported [22,67]. In brief, brains were fixed in neutral buffered formalin and embedded in 4% paraffin. Tissue was sectioned to a thickness of 3–5 µm by a microtome (Microm-hm 340e- BioOptica, Milan, It), and dried in the oven at 60 °C for one hour. Sections were stained with hematoxylin and eosin using an automated Leica ST5020 Multistainer (Leica Microsystem, Milano, It). The number of Purkinje cells (PCs) and the thickness of the external granule cell layer (EGL) were evaluated on three different fields, covering almost the whole Cll, by a D-sight plus image digital microscope and scanner (Menarini Diagnostics, Firenze, Italy). Data were expressed as mean ± S.D., and in fold vs. Normo (reference = 1).

### 4.4. Total Serum Bilirubin (TSB)

To monitor potential confounding effects of the drug challenge, the total serum bilirubin level was assessed in both Curc-treated pups and control animals. Blood samples were collected during the sacrifice, serum was separated by centrifugation (2000 rpm, 20 min RT) and the TSB was quantified by the diazo reaction, as previously described [67,85]. The results were expressed as mean ± S.D. and in mg/dL.

### 4.5. Behavioral Tests

Two tests were selected specifically for assessing the motor and coordination abilities of young rats, the righting reflex, and the negative geotaxis [86,87,88]. No training was required, and animals were asked to perform each test only one time a day to avoid fatigue. Since young rats still cannot perfectly keep their body temperature, one animal at a time was separated from the mother, tested, and returned to the mother. Each test does not require more than 2 min in total. The righting reflex was conducted at P9. The pups were gently placed on their back (supine position) on a comfortable surface, and the time required for them to roll on their stomach (prone position), was recorded. The negative geotaxis was performed at P11. The pups were gently placed on an inclined (30°) plane, with the head bottom-oriented. The time required for them to rotate 180° upside-down was recorded. Data were expressed as mean ± S.D. and in fold vs. Normo (reference = 1).

### 4.6. Real-Time PCR of Selected Markers for Inflammation, Redox Imbalance, and Brain Development

To follow the effect of bilirubin-induced brain damage and Curc protection, we monitored the genes (inflammation and redox state Table 1) previously reported [22], and chosen because they are known players in bilirubin neurotoxicity (see ref in the Introduction, in addition to [22]). For the Mbp analysis, we compared three different primer pairs (one designed by us, two from literature). Based on the tests (specificity, efficiency), we used the ones described by Abranches [89].

Total RNA extraction, retrotranscription, and RTqPCR were performed as previously described [22,67]. In brief, total RNA was extracted using TRI Reagent^®^ RNA Isolation Reagent (Sigma-Aldrich, St. Louis, MO, USA), and the complementary DNA (cDNA) was synthesized with the High Capacity cDNA Reverse Transcription Kit (Applied Biosystems, Monza, Italy), following the manufacturer’s instructions. The primers were designed using the Beacon Designer 4.2 software (Premier Biosoft International, Palo Alto, CA, USA) on rat sequences available in GenBank. The reaction was performed in an iQ5 Bio-Rad Thermal cycler (BioRad Laboratories, Hercules, CA, USA), in the SsoAdvanced SYBR green supermix (Bio-Rad Laboratories, Hercules, CA, USA). Amplification of target genes was accomplished using the following protocol: 3 min at 95 °C, 40 cycles at 95 °C for 20 s, 60 °C for 20 s, and 72 °C for 30 s. The specificity of the amplification was verified by a melting-curve analysis, and non-specific products of PCR were not found in any case. The relative quantification was made using the iCycleriQ software, version 3.1 (Bio-Rad Laboratories, Hercules, CA, USA) by the ΔΔCt method, taking into account the efficiencies of the individual genes and normalizing the results to the housekeeping genes [90,91]. Data were expressed as mean ± S.D., and in fold vs. Normo (reference = 1).

### 4.7. Glutamate Quantification

The amount of brain glutamate (Glut) was quantified by Glutamate Assay Kit following the producer’s instructions (MAK004, Sigma-Aldrich, MO) [22], with some adaptation. Briefly, Cll was mechanically homogenate by a Dounce potter in Glutamate Assay buffer (200 uL each 10 mg tissue), the sample was centrifuged at 13,000 g for 15 min, and the supernatant collected for performing the test. Glutamate content in Hyper and Curc samples was expressed as fold change compared to Normo, after normalization for the total protein content in each sample, quantified by the Bicinchoninic Acid kit following the supplier’s instruction (B9643 and C2284, Sigma-Aldrich, MO). Data were expressed as mean ± S.D., and in fold vs. Normo (reference = 1).

### 4.8. Protein Evaluation of Selected Markers for Inflammation, Redox Imbalance, and Brain Development

Western blot was performed as previously described [67] on 30 µg protein/well each animal/treatment (except for Hmox1 analysis that required 60ug protein/well each animal/treatment). In brief, Cll were mechanically homogenized and the protein concentration was determined by the Bicinchoninic Acid Protein Assay (B-9643 and C2284, Sigma, Missouri, USA). Thus, proteins were separated by 12% SDS-PAGE by electrophoresis in a Hoefer SE 250 System (Amersham BioSciences, UK), transferred onto immune-blot PVDF membranes (0.2 μm; Whatman Schleicher and Schuell, Dassel, Germany) at 100 V for 60 min (Bio-Rad Laboratories, Hercules, CA, USA). Blocking (1.5 h, RT), and incubation with the primary (Icam1 2 h RT; Hmox1 and Mag O/N, 4 °C) and secondary antibodies (2 h RT) was performed in 3% defatted milk in 0.2% Tween 20; 20 mM Tris-HCl pH 7.5; 500 mM NaCl. The details of the antibodies used are reported in Table 2. The signal was revealed by chemiluminescence (ECL-Plus Western blotting Detection Reagents, GE-Healthcare Bio-Science, Italy) and visualized on X-ray films (BioMax Light, Kodak Rochester, NY, USA). The results were normalized vs. the actin signal (1h RT), visualized incubating the same membrane. The band intensity was quantified by the Scion Image software (GE Healthcare Europe GmbH, France) [67].

Il1β and Tnfα level was quantified on tissues homogenates by ELISA kits (see Table 2), following the manufacturer’s instructions. The results were normalized for the total protein content in each sample, quantified by the Bicinchoninic Acid Protein Assay (B-9643 and C2284, Sigma, Missouri, USA). Data were expressed as mean ± S.D., and in fold vs. Normo (reference = 1).

### 4.9. Statistical Analysis

Data were analyzed with GraphPad Prism version 5.00 for Windows (GraphPad Software, La Jolla, CA, USA). Statistical significance was evaluated the one-way analysis of variance (ANOVA), followed by a Tukey–Kramer Multiple Comparisons Test when *p*-value < 0.05. A *p*-value < 0.05 was considered statistically significant.

## Figures and Tables

**Table 1:** Primers specifications.

Gene	Accession Number	Forward Primer	Reverse Primer	Amplicon Length (bp)
*Hprt*	NM_012583.2	AGACTGAAGAGCTACTGTAATGAC	GGCTGTACTGCTTGACCAAG	163
*Gapdh*	NM_017008.2	CTCTCTGCTCCTCCCTGTTC	CACCGACCTTCACCATCTTG	87
*Hmox1*	NM_012580.2	GGTGATGGCCTCCTTGTA	ATAGACTGGGTTCTGCTTGT	76
*Srxn1*	NM_001047858.3	AAGGCGGTGACTACTACT	TTGGCAGGAATGGTCTCT	85
*Tnfα*	NM_012675.2	CAACTACGATGCTCAGAAACAC	AGACAGCCTGATCCACTCC	172
*IL1β*	NM_031512.2	AACAAGATAGAAGTCAAGA	ATGGTGAAGTCAACTATG	137
*IL6*	NM_012589.1	GCCCACCAGGAACGAAAGTC	ATCCTCTGTGAAGTCTCCTCTCC	161
*Cox2*	NM_017232.3	CTTTCAATGTGCAAGACC	TACTGTAGGGTTAATGTCATC	92
*Icam1*	NM_012967	ACCTACATACATTCCTACC	ATGAGACTCCATTGTTGA	91
*Mag*	NM_017190	ACCATCCAACCTTCTGTATC	CTGATTCCGCTCCAAGTG	90
*Mbp*	NM_001025291	CACAGAAGAGACCCTCACAGCGACA	TCCATCGGGCGCTTCTTTAGCGG	150

*Hprt*: Hypoxanthine-guanine phosphoribosyl-transferase; *Gapdh*: Glyceraldehyde 3-phosphate dehydrogenase; *Hmox1*: Heme oxygenase1; *Srnx1*: Sulfiredoxin 1; *Tnfα*: Tumor necrosis factor alfa; *Il1β*: Interleukin 1β; *Il6*: Interleukin 6; *Cox2*: Cyclo-oxygenase 2; *Icam1*: intracellular adhesion molecule 1; *Mag*: myelin-associated glycoprotein; *Mbp*: myelin basic protein; bp: base pairs.

**Table 2:** Antibodies and ELISA kit specifications.

Target	Technique	Ab I Code and Dilution	Ab II Code and Dilution
Il1β	ELISA	ER2IL1B (Thermo Scientific, MA, USA)	
Tnfα	ELISA	Ab100785 (Abcam, ProdottiGianni, Milano, Italy)	
Actin	Western blot	A2066 (1:3000) (Sigma-Aldrich, Darmstadt, Germany)	Anti-Rabbit HRP (P0448; 1:3000) (Dako, CA, USA)
Hmox1	Western blot	Sc136961 (1:100) (SantaCruz, Aachen, Germany)	Anti-Mouse HRP (P0260; 1:3500) (Dako, CA, USA)
Icam1	Western blot	Sc1511 (1:50) (SantaCruz, Aachen, Germany)	Anti-Goat HRP (P0449; 1:4000) (Dako, CA, USA)
Mag	Western blot	Sc 166849 (1:1000) (MyBioSource, San Diego, CA, USA)	Anti-Rabbit HRP (1:3000) (Dako, CA, USA)
Mbp	Western blot	MBS175140 (1:100) (MyBioSource, San Diego, CA, USA)	Anti-Rabbit HRP (1:3000) (Dako, CA, USA)

*ELISA:* enzyme-linked immunosorbent assay. Ab: antibody. Il1β: Interleukin 1β; Tnfα: Tumor necrosis factor alfa; Hmox1: Heme oxygenase1; Icam1: intracellular adhesion molecule 1; Mag: myelin-associated glycoprotein, Mbp: myelin basic protein.

## Data Availability

The data presented in this study are available in the article.

## Abbreviations

Bf	free bilirubin
bp	base pairs
Cll	cerebellum
Curc	curcumin
EGL	external granular layer
ELISA	enzyme-linked immunosorbent assay
Glut	glutamate
*Hmox1*	heme oxygenase 1
Hyper	Hyperbilirubinemic Gunn pups
Hyper Curc	Hyperbilirubinemic Gunn pups treated with curcumin
*Icam1*	intracellular adhesion molecule
*Il1β*	interleukin 1 beta
i.p.	intraperitoneal injection
*Mag*	Myelin associated glycoprotein
*Mbp*	Myelin basic protein
Normo	Normobilirubinemic Gunn pups
Normo Curc	Normobilirubinemic Gunn pups treated with curcumin
P	post-natal age in days
PT	phototherapy
PCs	Purkinje cells
RTqPCR	Real-Time PCR
*Tnfα*	tumor necrosis factor alpha
TSB	Total serum bilirubin
KSD	kernicterus spectrum disorder
